# Functional MRI study in a case of Charles Bonnet syndrome related to LHON

**DOI:** 10.1186/s12883-019-1579-9

**Published:** 2019-12-30

**Authors:** V. Vacchiano, C. Tonon, M. Mitolo, S. Evangelisti, M. Carbonelli, R. Liguori, R. Lodi, V. Carelli, C. La Morgia

**Affiliations:** 1 IRCCS Istituto delle Scienze Neurologiche di Bologna, UOC Clinica Neurologica, Via Altura, 3, 40139 Bologna, Italy; 2 Unit of Neurology, Department of Biomedical and Neuromotor Sciences (DIBINEM), University of Bologna, Bologna, Italy; 3 IRCCS Istituto delle Scienze Neurologiche di Bologna, Diagnostica Funzionale Neuroradiologica, Bologna, Italy; 4 Functional MR Unit, Department of Biomedical and Neuromotor Sciences (DIBINEM), University of Bologna, Bologna, Italy; 5 Azienda Ospedaliero-Universitaria di Bologna, Bologna, Italy

**Keywords:** Charles bonnet, LHON, Functional magnetic resonance imaging (fMRI), Optic nerve

## Abstract

**Introduction:**

Charles Bonnet syndrome is characterized by simple or complex visual hallucinations (VH) due to damage along the visual pathways. We report a functional MRI study of brain correlates of VH in the context of a severe optic atrophy in a patient with Leber’s Hereditary Optic Neuropathy (LHON).

**Case report:**

A 62-year-old man was diagnosed with LHON (11778/ND4 mtDNA mutation) after subacute visual loss in left eye (right eye was amblyopic). One month later, he experienced VH of a few seconds consisting in “moving red and blue miniature cartoons”. One year later VH content changed in colored mosaic (10–15 s duration), usually stress-related, and blue and white flashes (2–5 s), triggered by unexpected auditory stimuli. Audiometry revealed mild sensorineural hearing loss. Three block design functional MRI paradigms were administrated: 1) random “clap”, 2) “checkerboard” and 3) non-random “beep”. After random “claps” simple flashes were evoked with bilateral activation of primary and secondary visual cortex, cuneus, precuneus and insula. Neither hallucinations nor cortex activation were registered after “checkerboard” stimulation, due to the severe visual impairment. Primary and secondary auditory cortices were “beep”-activated, without eliciting VH by non-random “beep”.

**Conclusions:**

The peculiarity of our case is that VH were triggered by random auditory stimuli, possibly due to a cross-modal plasticity between visual and auditory networks, likely influenced by the sensorineural deafness. Functional alterations of both networks in resting conditions have been demonstrated in LHON patients, even without an auditory deficit. Finally, the absence of VH triggered by expected stimuli is consistent with the “expectation suppression theory”, based on increased neural activations after unexpected but not by predicted events.

## Introduction

Charles Bonnet syndrome (CBS) is a condition characterized by the presence of visual hallucinations (VH) due to damage along the visual pathways, in the absence of cognitive deficits or psychiatric disorders. VH can vary from simple images, such as flashes, shapes, grid-like patterns, or complex images such as people, faces, animals and objects. The pathophysiology of CBS is likely related to mechanism of denervation hypersensitivity caused by sensory deprivation.

## Case report

A 62-year-old right-handed man was diagnosed with Leber’s Hereditary Optic Neuropathy (LHON, m.11778G > A/ND4 mtDNA mutation) after subacute visual loss in the left eye (LE) (right eye [RE] was amblyopic). One month later, he experienced VH lasting a few seconds consisting in “moving red and blue miniature cartoons”. At that time visual acuity was hand motion (HM) in RE and counting fingers (CF) in LE, whereas color vision was 0/12 at Ishihara plates. Computerized visual fields showed a generalized defect in RE and a pseudo-altitudinal defect in LE (Additional file 1: Figure S1). One year later VH content changed in colored mosaic (10–15 s duration), usually stress-related, and blue and white flashes (2–5 s), triggered by unexpected auditory stimuli. Audiometry revealed mild sensorineural hearing loss.

To obtain a detailed description of VH a semi-structured interview was performed prior and after the functional Magnetic Resonance Imaging (fMRI) study [1].

Three block-design functional MRI paradigms were administrated using a 1.5 Tesla GE Medical Systems Signa HDx 15 system: 1) random “clap” (six claps with the same intensity, duration 10 min), 2) “checkerboard” (visual stimulation, duration 5 min), 3) non-random “beep” (generated through the software “Audacity”, duration 5 min).

**Fig. 1:**
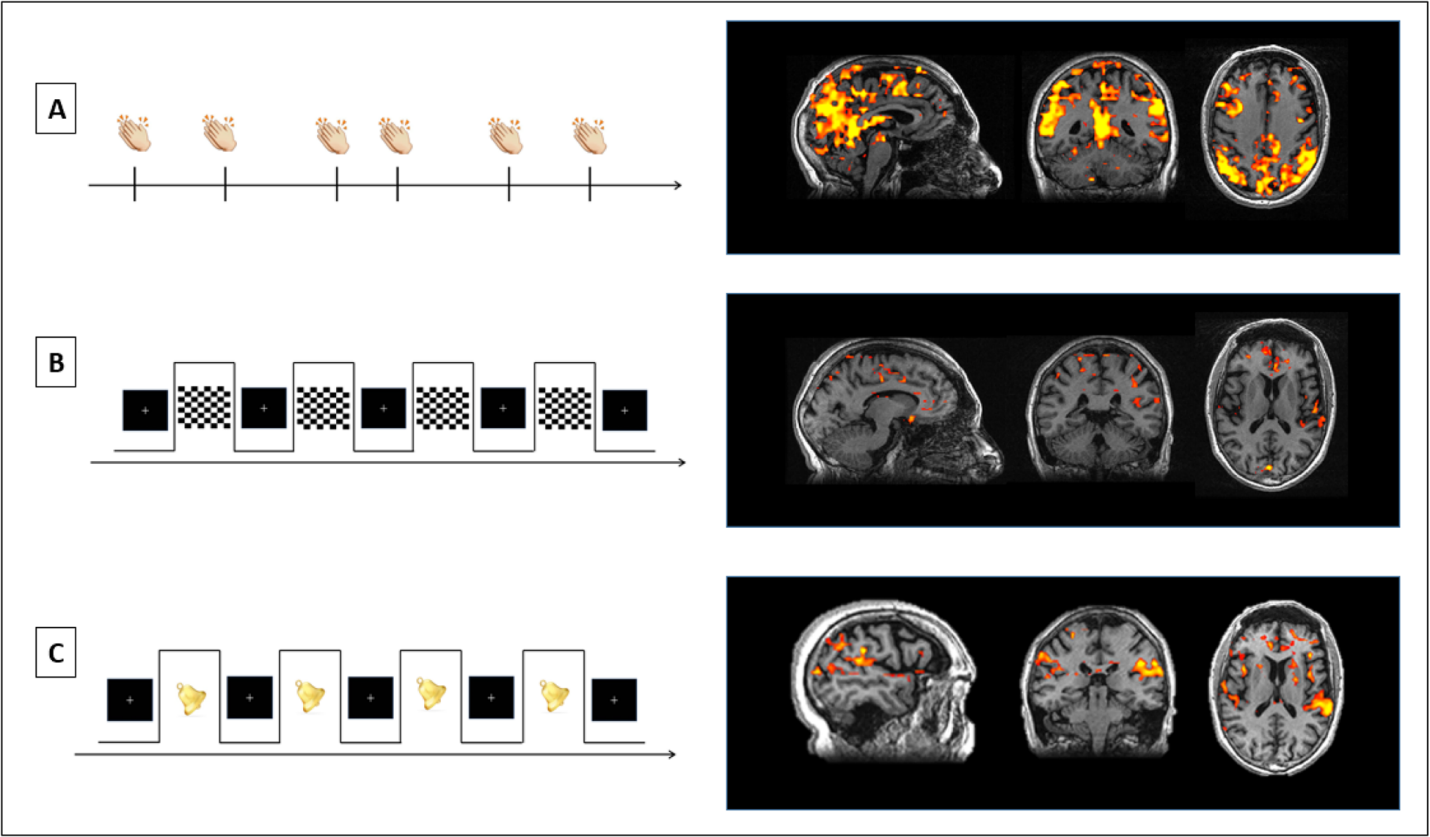
fMRI study in Charles Bonnet syndrome related to LHON. **a** Clap random stimuli: bilateral activation of primary and secondary visual cortex, cuneus, precuneus and insula (five flashes reported) in LHON patient. **b** Checkerboard: no activation of visual cortices (VH not reported) in LHON patient. **c** Non-random beep: activation of primary and secondary auditory cortex (flashes not reported, only stress-related colored mosaic reported) in LHON patient

Claps and beeps were administrated through MR compatible earphone that isolate from the background MRI noise. Moreover, the MRI noise was constant during the fMRI acquisition, therefore did not influence the GLM design. In addition, in order to familiarize with the MRI noise, patient underwent first structural sequences followed by fMRI paradigms. The patient was trained to report the onset of each VH by pressing a handgrip (NNL, NordicNeuroLab). Random clap stimuli evoked five simple VH (flashes) with bilateral activation of primary and secondary visual cortex, cuneus, precuneus and insula, consistent with previous fMRI findings (Fig. 1a) [2]. Neither hallucinations nor visual cortices activation were observed after checkerboard stimuli, consistently with the severe visual loss and optic atrophy (Fig. 1b). Finally, non-random beep activated the primary and secondary auditory cortex eliciting stress-related VH (i.e. colored mosaic) without inducing “flashes” (VH), which were instead triggered by unexpected auditory stimuli (Fig. 1c).

## Discussion and conclusions

The pathophysiology of CBS is usually explained by the deafferentation hypothesis (also called sensory deprivation theory), which suggests that lack of sensory visual input causes spontaneous neuronal discharge and increases excitability within the visual association cortex.

It has been known that people with dual sensory loss in vision and hearing are more likely to suffer from CBS, thus reinforcing the deafferentation theory as cause of this condition [1].

CBS in LHON has been already reported in ten patients from a cohort of 190 LHON [3] and, previously, in another case, likely related to the administration of topical brimonidine, a lipophilic α-2 agonist that may cause central nervous system side effects in blind patients, probably in relation to the activation of hypersensitive and hyperexcitable neurons of the occipital lobe [4]. This is the first fMRI study on CBS in LHON.

Previous fMRI studies in patients with CBS demonstrated hyperactivity of the ventral occipital lobe during VH, with specific correlation between the location of activity within specialized cortex and the content of the visual hallucinations [2].

The peculiarity of our case is that VH were triggered by random auditory stimuli (“clap stimuli”). This is an example of acquired auditory-visual synesthesia (AVS), a phenomenon whereby auditory stimulation triggers visual experience. A possible mechanism of acquired AVS involves cross-modal plasticity between visual and auditory networks after a sensory modal loss, due to disinhibition of the pre-existing pathways connecting primary sensory cortices [5]. The pre-existence of these networks is assumed since AVS often occurs shortly after the sensory deprivation. This hypothesis is reinforced by the “sound-induced flash illusion” [6], a paradoxical phenomenon where the auditory stimulation influences the visual cortical excitability even in healthy subjects, confirming the presence of physiological networks between the two areas.

Previous fMRI studies [5] have demonstrated the activation of the occipital lobe during sound-induced visual experience in patients with acquired AVS, as also found in our patient, probably facilitated by the presence of mild deafness. Indeed, hearing loss could cause an increased neural responsiveness of the auditory cortex to visual and/or tactile stimuli, due to the same pathological mechanism. This has been previously demonstrated by fMRI studies confirming changes in functional connectivity between auditory and visual sensory areas (mainly middle temporal visual area which is involved in visual motion processing), even in older patients with high-frequency hearing loss [7]. Functional and structural alterations of visual and auditory networks in resting conditions have been already demonstrated in LHON patients, even without deafness, corroborating the notion of a cross-modal plasticity between these sensory modalities also in this disorder [8].

Finally, the absence of VH triggered by expected stimuli (“beep”) and the reduced extent of brain activation during this paradigm is consistent with the “expectation suppression theory”, as confirmed by fMRI studies showing larger neural activations for surprising events in comparison to the neutral and correctly predicted conditions for alternating trials [9].

## Supplementary information

**Additional file 1: Figure S1.** Computerized visual fields: generalized defect in RE (A) and pseudo-altitudinal defect in LE (B) (PPTX 226 kb)

## Data Availability

Not applicable.

## Abbreviations

AVS: Auditory-visual synesthesia
CBS: Charles Bonnet syndrome
fMRI: Functional magnetic resonance imaging
LHON: Leber’s Hereditary Optic Neuropathy
NNL: NordicNeuroLab
VH: Visual hallucinations
