# Supplementary figures and images for: Functional MRI study in a case of Charles Bonnet syndrome related to LHON
Source: BMC Neurol. 2019 Dec 30;19:350. doi: 10.1186/s12883-019-1579-9 (PMC6937628; doi:10.1186/s12883-019-1579-9)

## Slide 1
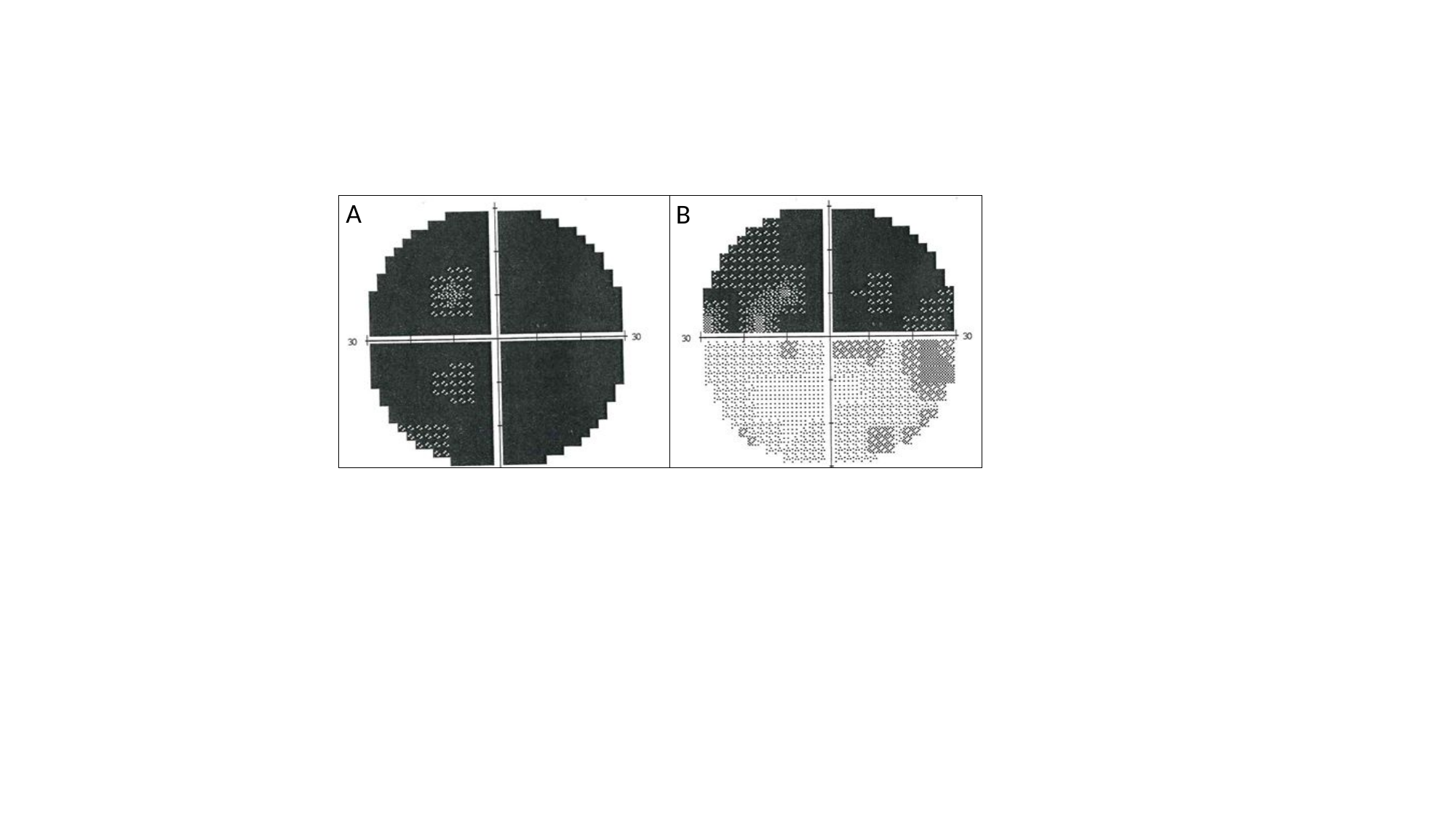

A
B

Supplement: Supplementary file 1 — Additional file 1: Figure S1. Computerized visual fields: generalized defect in RE (A) and pseudo-altitudinal defect in LE (B) (PPTX 226 kb) [file 12883_2019_1579_MOESM1_ESM.pptx]
